# Design and Construction of a Multi-Tiered Minimal Actin Cortex for Structural Support in Lipid Bilayer Applications

**DOI:** 10.1021/acsabm.3c01267

**Published:** 2024-03-01

**Authors:** Amanda J. Smith, Theodore R. B. Larsen, Harmony K. Zimmerman, Samuel J. Virolainen, Joshua J. Meyer, Lisa M. Keranen Burden, Daniel L. Burden

**Affiliations:** Chemistry Department, Wheaton College, 501 College Ave., Wheaton, Illinois 60187, United States

**Keywords:** lipid bilayer, actin, reconstitution, biomembrane, nanopore Biosensors, layer-by-layer, polyelectrolyte, surface dynamics

## Abstract

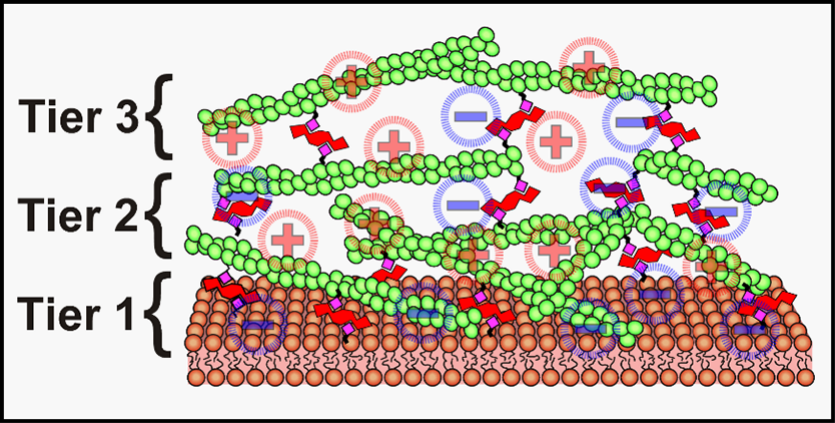

Artificial lipid bilayers have revolutionized biochemical and biophysical research by providing a versatile interface to study aspects of cell membranes and membrane-bound processes in a controlled environment. Artificial bilayers also play a central role in numerous biosensing applications, form the foundational interface for liposomal drug delivery, and provide a vital structure for the development of synthetic cells. But unlike the envelope in many living cells, artificial bilayers can be mechanically fragile. Here, we develop prototype scaffolds for artificial bilayers made from multiple chemically linked tiers of actin filaments that can be bonded to lipid headgroups. We call the interlinked and layered assembly a multiple minimal actin cortex (multi-MAC). Construction of multi-MACs has the potential to significantly increase the bilayer’s resistance to applied stress while retaining many desirable physical and chemical properties that are characteristic of lipid bilayers. Furthermore, the linking chemistry of multi-MACs is generalizable and can be applied almost anywhere lipid bilayers are important. This work describes a filament-by-filament approach to multi-MAC assembly that produces distinct 2D and 3D architectures. The nature of the structure depends on a combination of the underlying chemical conditions. Using fluorescence imaging techniques in model planar bilayers, we explore how multi-MACs vary with electrostatic charge, assembly time, ionic strength, and type of chemical linker. We also assess how the presence of a multi-MAC alters the underlying lateral diffusion of lipids and investigate the ability of multi-MACs to withstand exposure to shear stress.

## Introduction

The actin cytoskeleton drives morphogenic changes in the cell and helps stabilize the membrane shape. Actin also preserves many of the unique chemical and physical properties of the lipid bilayer. These combined functions make actin appropriate for applications in which the molecular properties of lipids and mechanical stability are important. Artificial supports for bilayers mimic the role of the cellular cytoskeleton and are employed throughout biophysics, biomaterials, and bioengineering research, offering a platform for studying membrane properties, cell signaling, membrane–protein interactions, and bilayer functionalization.^1−12^ Numerous strategies have been developed, such as forming bilayers on hydrated polymer cushions,^13^ tethering the bilayer to solid surfaces^14^ or microcavities,^15^ trapping the bilayer between two hydrogel layers,^16^ photopolymerizing reactive amphiphiles in the lipid membrane,^17^ cross-linking lipid molecules comprising the bilayer,^18^ and polymerizing actin within liposomes.^19^

A number of emerging sensing technologies critically depend on robust lipid bilayers, including those based on biological nanopores.^20−26^ To work properly, analytes must be able to move from the bathing solution surrounding the interface to the mouth of the nanopore. Specific nanopore sensing applications include DNA sequencing,^27^ RNA sequencing,^28−30^ nucleic acid detection,^31−33^ polypeptide detection,^34^ RNA profiling,^35^ synthetic polymer characterization,^36−38^ digital data storage,^39−41^ disease detection,^42^ ion sensing,^43^ small molecule detection,^44^ and sensing of protein–drug interactions.^45^ Multiple types of nanopores with various pore sizes and channel structures can be employed. Because diffusive access to the bilayer (and lateral diffusion within the bilayer) are critical for sensing applications, bulky structures added for mechanical support can slow, or block, movement to and from, as well as lateral movement within, the lipid interface.^17^ The thin, filamentous, and porous nature of the actin network is well-suited for both support and rapid penetrability, assuming the layered network can be successfully constructed on a bilayer.

Previously, we developed an approach for modifying the interface of free-standing planar lipid membranes with a tethered single layer of filamentous actin (F-actin) linked to the bilayer using biotin and streptavidin. We observed enhanced resistance to mechanical stress and noted that diffusive access to the bilayer was not inhibited.^46^ Similar synthetic actin structures have been developed by others and are referred to as a minimal actin cortex (MAC).^47−50^ MACs have been primarily developed for *in vitro* studies of actin–myosin membrane interactions and the exploration of cortex mechanics.^51^ Here, we explore the possibility of synthesizing multiple distinct cross-linked tiers of actin on a lipid bilayer as a united multilayered and bonded structure (dubbed a multi-MAC) that can potentially convey added strength and stability to a wide array of bilayer structures.

This work describes a filament-by-filament approach to creating multi-MACs with various 2D and 3D architectures. We utilize total internal reflection fluorescence (TIRF) and widefield fluorescence imaging on glass-supported planar bilayers to explore various modes of forming multi-MACs in distinct molecular layers. Fluorescence recovery after photobleaching (FRAP) enabled lateral lipid diffusion characterization. More specifically, we (1) demonstrate a range of achievable 2D and 3D filament densities in single-tiered MACs; (2) describe the effects of electrostatic forces and ionic strength on the creation of multi-MACs that employ a variety of linkers from the avidin family; (3) reveal the approximate time scales required for filament-by-filament deposition; (4) confirm the feasibility of constructing multiple stacked and cross-linked tiers of F-actin (i.e., a multi-MAC); (5) address the translational diffusion of lipids when a MAC is linked to the bilayer; and (6) demonstrate the resistance of multi-MACs to shear stress in a flowing aqueous stream.

Given a method to construct numerous thin tiers, multi-MACs possess the potential for enhancing resistance to mechanical and electrical stress in ways that exceed the original single-layer study. Capitalizing on the unique combination of strength, size, and morphology of multi-MACs can assist many burgeoning biotechnological applications that depend on robust lipid bilayer partitions for their success.

## Materials and Methods

### Fluorescence Microscopy

Samples were interrogated by a Nikon Eclipse T*i* microscope equipped with a TIRF illuminator arm and a Perfect Focus drift compensation system. TIRF illumination excites fluorescence in a thin region above the glass–water interface (∼500 nm). The TIRF arm allowed control of the laser angle through the objective so that imaging modes could be switched between TIRF and widefield illumination at will. Widefield imaging probes more deeply into the sample and permits characterization of free actin filaments in solution above the surface as well as filaments located away from the interface. The TIRF illuminator was connected to a single-mode fiber optic that delivered excitation light from a 532-nm laser. The power output through the objective (Nikon 100×/NA 1.49, oil immersion) in the widefield mode was ∼1.5 mW, which corresponds to an intensity of ∼35 W/cm^2^. Fluorescence was detected by using a bandpass filter with a 565–595 nm transmission window. Images and videos were collected by an EMCCD camera (Andor, iXon) under the control of Nikon Elements software.

### TIRF Flow Cell Sample Chamber Preparation

Supported lipid bilayers (SLBs) were formed on borosilicate glass coverslips. First, the coverslips (15 mm round, No.1, Warner Instruments) were cleaned by sonicating for 1 h in sodium dodecyl sulfate (1 g SDS per 300 mL ultrapure water) and sonicating for another hour in reagent grade isopropanol. The cleaned slides were then rinsed and stored in ultrapure water (18.2 MΩ) until use. Immediately before use, the coverslips were dried by using the flame tip from a Bunsen burner. Coverslips were then placed in the flow cell chamber (RC-25F, Warner Instruments), which produces a Laminar flow over the surface by a gravity-driven perfusion system in an ∼1 mL chamber.

### Small Unilamellar Vesicle (SUV) and SLB Preparation

1,2-dioleoyl-*sn*-glycero-3-phosphate (DOPC) and 1,2-Distearoyl-*sn*-Glycero-3-Phosphoethanolamine-N-[Biotinyl(Polyethylene Glycol)2000] (ammonium salt) (DSPE-PEG(2000)Biotin) were purchased from Avanti Polar Lipids (Alabaster, AL). N-(Tetramethylrhodamine-6-thiocarbamoyl)-1,2-dihexadecanoyl-*sn*-glycero-3-phosphoethanolamine and triethylammonium salt (TRITC-DHPE) were purchased from Biotium (San Francisco, CA). All lipids were dissolved in chloroform and mixed to form solutions with varying percentages of biotinylated or fluorescently labeled lipids (e.g., 0–1 mol % labeled:unlabeled lipid).

Lipid solutions were dried under a nitrogen stream to evaporate the chloroform (approximately 20 min for a 1 mL solution). While drying, the 2-mL glass vials were held at a 45° angle and gently rotated to facilitate a uniform deposition of lipids over a large surface area within the vial. The lipids were further dried under vacuum for 1 h. The dried lipids were resuspended by vortexing in a 10-mM Tris–HCl buffer, pH 7.5, at a lipid concentration of 2.5 mg/mL. The resulting turbid suspensions were then sonicated to clarity (5–10 min) to form SUVs.

Prior to addition, an aliquot of the SUV solution was diluted to 0.5 mg/mL, and 200 μL was then added to the cleaned cover glass in the perfusion chamber. The solution was incubated for 15 min to allow vesicles to fuse, rupture, and cover the glass surface in a bilayer. Residual lipids were rinsed from the chamber using 10 mM Tris–HCl (10–15× volume exchange). The effectiveness of this procedure for creating SLBs was assessed via TIRF imaging to verify the uniformity and FRAP to ensure that the lipid surface was fluid.

### Polymerized Actin Filament Preparation

Rabbit skeletal actin monomers >99% pure (Cat: AKL99), biotinylated actin monomers (AB07), and rhodamine-labeled actin monomers (AR05) were ordered from Cytoskeleton, Inc. and mixed to form solutions with a mole ratio of 4:1:1, respectively. The lyophilized monomers were resuspended and diluted to 2 μM in a buffer of 0.2 mM CaCl_2_ and 5 mM Tris–HCl pH 7.5. The actin solutions were left on ice for 1 h to promote depolymerization. Remaining nucleation centers were removed by centrifuging for 30 min at 4 °C and 16873 × *g* and using the top 80% of the supernatant. To initiate polymerization, concentrated polymerization buffer was added to obtain final conditions of 10 mM Tris HCl, 2 mM MgCl_2_, 50 mM KCl, 1 mM ATP, and 5 mM guanidine carbonate at pH 7.5. To stabilize filaments and lower the critical concentration required for polymerization, phalloidin was added at the start of the polymerization process in a 10-fold molar excess to the actin monomers. Actin filaments were left at room temperature for 1 h to polymerize and were then stored at 4 °C until needed for experiments.

### Linker Deposition

Three different linkers were explored for multi-MAC construction: avidin (Sigma-Aldrich, A9275), streptavidin from *Streptomyces avidinii* (Sigma-Aldrich, S4762), and neutravidin (ThermoFisher, 31000). Avidin, streptavidin, and neutravidin have similar aggregate structures, possessing four biotin binding sites per tetrameric aggregate and a monomer molecular weight of ∼67, ∼53, and ∼60 kDa, respectively. Avidin possesses numerous glycosylation sites over its surface; streptavidin lacks glycosylation. Neutravidin also lacks glycosylation and has been additionally engineered to remove RYD recognition sequences in order to minimize nonspecific binding. The three linkers possess a similar biotin-binding affinity with dissociation constants on the order of 10^–15^ M.^52,53^ A significant difference between the linkers is the isoelectric point, with values of 5.0 for streptavidin,^52^ 6.3 for neutravidin,^54^ and 9.5 for avidin.^55^ Electrostatic fields generated by the linkers at pH 7.5 impact the layering dynamics and the resulting 3D structure of the F-actin. Linkers were prepared for single-MAC and multi-MAC construction by dilution in 10 mM Tris–HCl, pH 7.5, to a stock concentration of 0.001–0.01 mg/mL. The concentration chosen was dependent upon biotin concentration with the intention of saturating all available biotin binding sites. Typically, 200 μL of stock was added to the 1 mL flow cell for a final concentration of ∼0.0002 to 0.002 mg/mL (30–400 nM), which was gently mixed and incubated before perfusing the chamber free of additives with ∼10 mL (10× chamber volume) of buffer.

### Actin Filament Deposition and Layering

**Figure 1:**
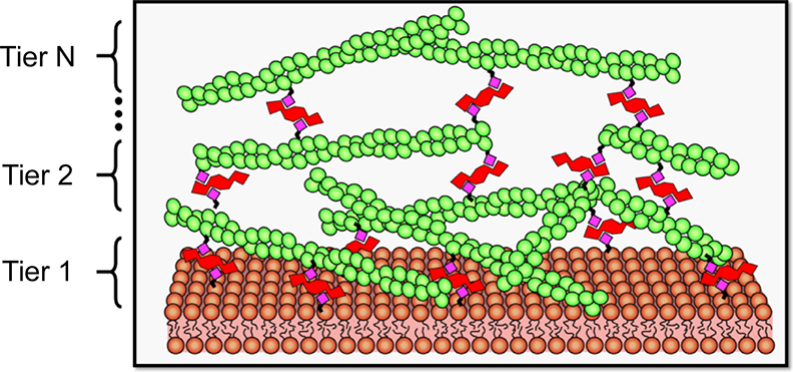
Multi-MAC construction involves iterative application of F-actin and linkers following an (A-wash-B_*n*_-wash)_*N*_ procedure. Biotin moieties are depicted as pink diamonds. Linkers, each with four biotin-binding sites, are depicted in red. Actin filaments are in green. An arbitrary number (*N*) of F-actin tiers can be formed.

Multi-MAC creation followed an iterative (A-wash-B_*n*_-wash)_*N*_ algorithm, where A designates a saturating linker deposition step, B designates F-actin deposition, *n* denotes the number of incremental F-actin additions required to reach surface saturation, and *N* indicates the number of connected F-actin tiers (Figure 1). Wash steps were executed to remove residual unbound material from the deposition chamber after the addition of linker (A) and F-actin (B_*n*_), respectively. Washes consisted of 5–10× chamber volume exchanges using solutions of 10 mM Tris–HCl (pH 7.5) in KCl (0 mM–1 M). The addition of a salt enabled control of the electrostatic interactions between molecules. Low ionic strength conditions were created in 10 mM Tris–HCl (pH 7.5) and 0–50 mM KCl. High ionic strength conditions consisted of 10 mM Tris–HCl at pH 7.5 and 0.5–1 M KCl.

After the formation of an initial biotinylated bilayer from SUVs, linkers were added to the biotin sites. Following the wash step to remove excess linker, multiple aliquots (*n*) of biotinylated F-actin were delivered. For many experiments, multiple actin additions were performed until the apparent surface density of filaments no longer increased (i.e., *n* = 2–5). A second wash step was then performed to remove any unbound F-actin. This (A-wash-B_*n*_-wash) process was repeated multiple times (N) until the desired number of linked F-actin tiers was achieved. In this work, we explored up to *N* = 3 tiers. But, in principle, the upper limit to N is arbitrary.

We injected F-actin into the flow cell at low concentrations in order to deposit filaments in an incremental fashion. Truncated pipet tips were used whenever polymerized actin was transferred in order to minimize filament cleavage due to shear forces generated in the tips. A gradual filament-by-filament deposition process helped control the surface density, layer thickness, exposure to cross-linking sites, filament directionality, structural porosity, and mechanical rigidity. But most importantly for this study, it provided a means to visualize the MAC assembly process (see the Supporting Information for video). Accelerated deposition using concentrated F-actin solutions often resulted in uneven deposition and the presence of entangled actin masses. Depending on the experiment, aliquots of actin filaments were diluted from the polymerized stock by 4–40-fold. This dilution step permitted the transfer of a larger volume of F-actin into the chamber (∼200 μL). Larger volumes of diluted filaments promote a more even spatial distribution throughout the solution and avoid the undesirable deposition of entangled clumps. In some instances, actin filaments were allowed to incubate for up to 90 min before the chamber was rinsed. This duration is considerably longer than the incubation time necessary for filament binding but was primarily used to assist with image acquisition.

### SOAX Image Analysis

Actin filament images were processed using SOAX,^56^ an open-source program which uses a stretching-open-active-contours algorithm to trace the centerline of filaments. Output from the program includes lengths of filaments and numbers of filaments, which were used to compute filament density. Since MACs and multi-MACs are relatively thin, the filament density terminology used here refers to the number of filaments per square micrometer of the acquired image.

### FRAP

FRAP was used to determine the lateral diffusion of the lipids in the bilayer. To obtain an initial reference of the fluorescence intensity, a series of images was collected from a 75-μm diameter spot using TIRF illumination. The bilayer was illuminated for 50 ms once every second using a low illumination intensity (∼35 mW/cm^2^). The laser was then adjusted to full intensity (35 W/cm^2^) for a period of 10 s in order to photobleach the bilayer. Following the bleach, the diffusive recovery of the fluorescent lipids was monitored by illuminating the bleached spot at low excitation intensity (∼35 mW/cm^2^) for 50 ms each second and acquiring an image every second over a 20-min recovery period. The average intensity from the images was then normalized to the reference and plotted versus time for analysis.

### Fluorescent Bead Velocity for Sheer Stress Quantification

Chamber perfusion was driven by the gravitational flow of the buffer from an elevated reservoir. Because of this, the laminar flow velocity over the muti-MAC surface was controlled by the difference in elevation of the stock buffer solution and the perfusion chamber. After the formation of the desired MAC using fluorescently labeled actin, the stock buffer elevation was set, and the actin surface was rinsed. Photos collected before and after exposure to the flowing liquid allowed changes in the multi-MAC structure during the rinse to be assessed. To calculate the rinse velocity, 0.04-μm carboxylate-modified microspheres (FluoSpheres) from molecular probes were added while rinsing, and a video recorded the bead location across the surface. Using the relative position of individual beads between frames along with the elapsed time allowed for an average velocity to be calculated. Rinse velocities of ∼50 μm/s were typically used. Alterations to the MAC structure were tested at velocities of up to ∼325 μm/s.

## Results and Discussion

### Filament Length Distribution

**Figure 2:**
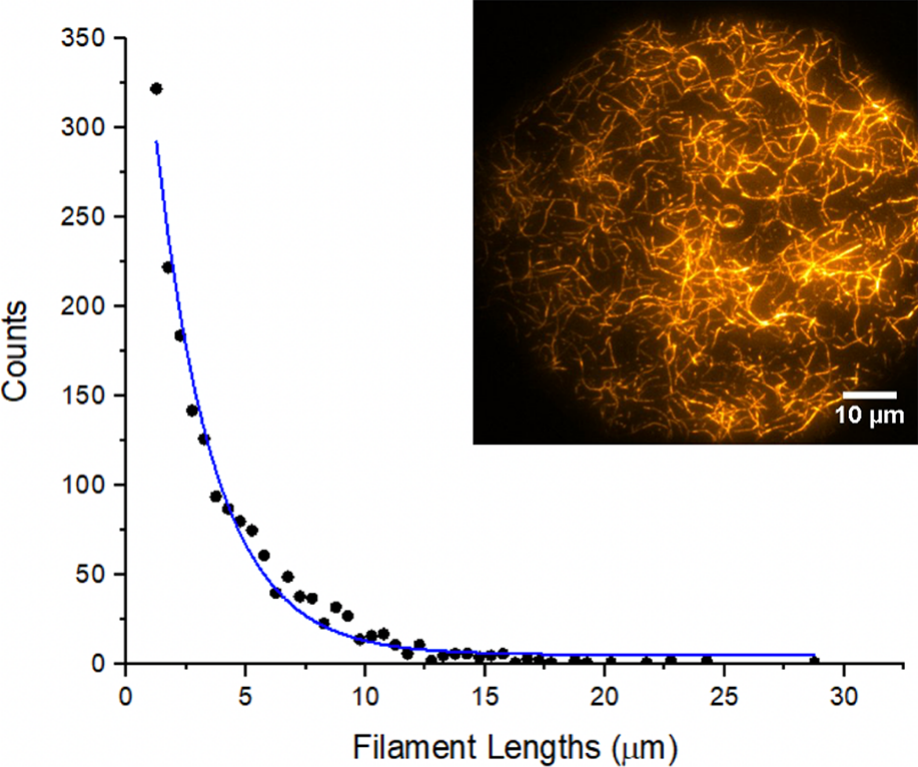
A single MAC constructed using 1 mol % biotinylated lipids and neutravidin linker in the presence of a 0.5 M KCl with a 10-min incubation. SOAX analysis reveals an exponential length distribution, which is typical for all linkers under optimal deposition conditions.

Individual filaments possess varying lengths that are determined by several parameters, such as ATP availability, actin monomer concentration, phalloidin availability, and various reaction conditions. A SOAX analysis of full-length horizontally oriented filaments reveals that our procedure favors an exponential length distribution with an average of ∼2.4 μm. Models of actin polymerization predict similar distributions.^57^Figure 2 shows a typical image of bilayer adherent filaments and the corresponding output from a SOAX analysis. Because the diffraction limit of the apparatus is ∼500 nm, the length of the short filaments cannot be distinguished from individual fluorescent molecules. In an effort to ensure that only polymerized actin molecules contribute to the distribution, we discard filaments that measured less than 1 μm. The resulting lengths are comparable to F-actin strands found within biological cells, where typical sizes range from 100 nm to a few microns.^58^

Filament binding and extension follow a dynamic process whereby unbound portions of a strand adhere in a gradual end-to-end sequence over time (see Supporting Information Video S1). We observed a similar sequential binding process for all linkers tested. Although the time scale to reach equilibrium and the final length distribution varies widely with linker type and deposition conditions, all linkers give roughly the same distribution under conditions that promote full lengthwise adherence. Thus, the data from Figure 2 are representative of all linker types and all biotin anchor concentrations.

**Figure 3:**
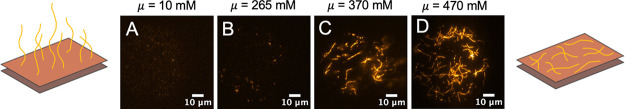
Electrostatic interactions dictate filament orientation and can be controlled by salt screening. F-actin (positively charged) displays less repulsion for streptavidin-coated bilayers (also positively charged) as the ionic strength (μ) increases. This causes filaments to rearrange from a tethered vertical orientation (A, B) to an adherent horizontal layer (C, D). All images were acquired after a 10-min incubation period.

We discovered that the equilibrium length distribution depends on the ionic strength employed during the deposition. For example, when a streptavidin linker is employed in the presence of a buffer with very low ionic strength, we observe virtually no adherent filaments (Figure 3A). As the ionic strength is increased, fluorescent spots become visible, which suggests the binding of very short filament segments (Figure 3B). As the ionic strength is further increased, these short segments appear to grow longer and extend within the plane of the bilayer (Figure 3C), as the remaining length of the filament adheres to the membrane. At high ionic strength, the full contour length of the filaments binds to the lipid membrane (Figure 3D). We attribute this change in adherence length to electrostatic interactions between the linker (streptavidin) and the F-actin.

The avidin family of linkers (i.e., streptavidin, neutravidin, and avidin) possesses isoelectric points (pIs) of 5, 6.3, and 9.5, respectively. We exclusively used streptavidin isolated from *Streptomyces avidinni*, which has a significantly lower pI than recombinant streptavidin. Actin isolated from rabbit skeletal muscle has a reported pI of ∼4.8.^59^ Thus, at pH 7.5, both actin and streptavidin bear a negative charge. This results in an electrostatic repulsion of the filaments from the surface and prohibits many F-actin molecules from attaching to the bilayer (Figure 3A). Furthermore, most filaments adhere to the surface only at one location within the polymer strand. These tethered filaments appear short due to the nature of TIRF illumination. A majority of the strand extends away from the bilayer in an orthogonal direction (Figure 3, left illustration) and cannot be visualized by TIRF. Evidence for vertical filament orientation can be seen when the illumination mode of the microscope is changed from TIRF to widefield. Because widefield imaging permits visualization above the surface (at the expense of image contrast), anchored but orthogonally extended filaments can be seen wavering in solution above the bilayer (see Supporting Information, Video S2). At higher salt concentrations, the repulsion between actin and streptavidin weakens, which allows a greater portion of the filament’s contour length to attach to the bilayer. Thus, the apparent length distribution of the imaged filaments grows larger. At the highest ionic strength, salt effectively screens the repulsive charges and filaments are permitted to fully adhere with a horizontal orientation (Figure 3, right illustration).

**Figure 4:**
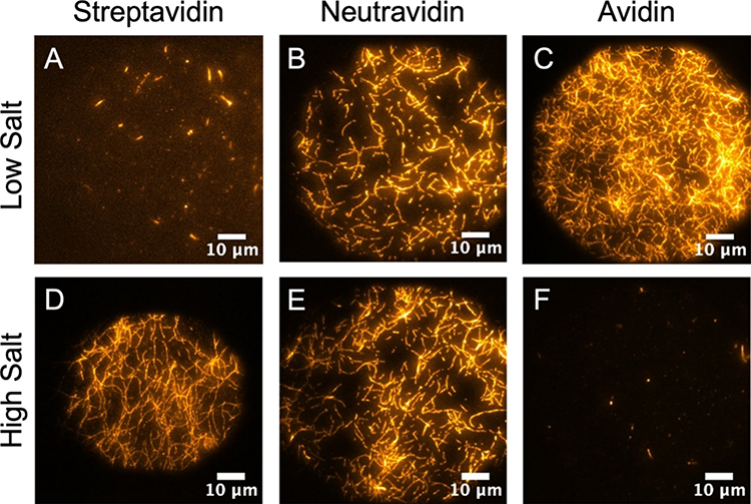
Filament binding at high and low salt concentration (1 M and 50 mM KCl) as a function of linker type after a 10-min incubation period: (A, D) streptavidin; (B, E) neutravidin; (C, F) avidin. Electrostatic interactions, as governed by the pI of each component, control the nature of binding (see text).

The impact of electrostatic interactions on the apparent filament length and orientation can be seen in Figure 4. Under low salt conditions (50 mM KCl) negative charge on both the linker and F-actin is not screened effectively. This charge governs intermolecular interactions and the deposition process. Figure 4A indicates a lack of F-actin binding because both streptavidin and F-actin are similarly charged. Thus, repulsion leads to sparce filament binding and the appearance of short filaments on the surface. This interpretation is further substantiated by a video that captures the transition from TIRF to widefield imaging without salt present (0 mM KCl). The video shows little binding to the lipid bilayer but many long filaments are freely hovering a short distance above the surface (see the Supporting Information, Video S3). Neutravidin (Figure 4B), with a near neutral pI, appears relatively unaffected by the lack of charge screening. Thus, F-actin more readily deposits with a horizontal orientation, albeit moderately. Similar results would be expected for recombinant streptavidin, which has a pI closer to 7. Figure 4C shows that the attraction between avidin (positively charged) and F-actin (negatively charged) readily pulls filaments to the surface and promotes abundant horizontal binding.

Under high salt concentrations (1 M KCl), we observe a different trend. Figure 4D indicates that high salt conditions effectively screen the electrostatic repulsion between F-actin and streptavidin and promote horizontal binding of the full filament contour length. Figure 4E indicates that neutravidin is again relatively unaffected by charge screening due to its near-neutral pI; therefore, full horizontal deposition is permitted. Lastly, Figure 4F shows very little binding, which seems to suggest that charge screening negates the attractive interactions between positively charged avidin and negatively charged F-actin. This result is counterintuitive. Electrostatic screening should not generate the functional equivalent of repulsion. Nonetheless, little filament binding is observed, and most F-actin strands appear to undulate in solution just above the bilayer (similar to Video S3). We postulate this apparent repulsion is due to the unique effect of avidin’s surface glycosylation. The presence of carbohydrate polymers attached across the linker protein surface adds a steric barrier to the approach of F-actin. Apparently, the steric interference can be overcome when the attractive interaction is large (i.e., conditions illustrated in Figure 4C). But when the innate charge of both proteins is lessened by the high salt concentration, the reduced attractive force is insufficient to overcome the steric barrier created by the conjugated carbohydrates. Thus, little F-actin binding is observed. Time-lapse TIRF video evidence from a continuous experiment involving fluid exchange supports this interpretation. In the video, solutions are alternated back and forth from low to high salt concentration; F-actin binding to the surface responds directly to the changing electrostatic conditions as well as the steric barrier present in avidin (see Supporting Information, Video S4).

### Impact of Linker on Filament Deposition Rate

**Figure 5:**
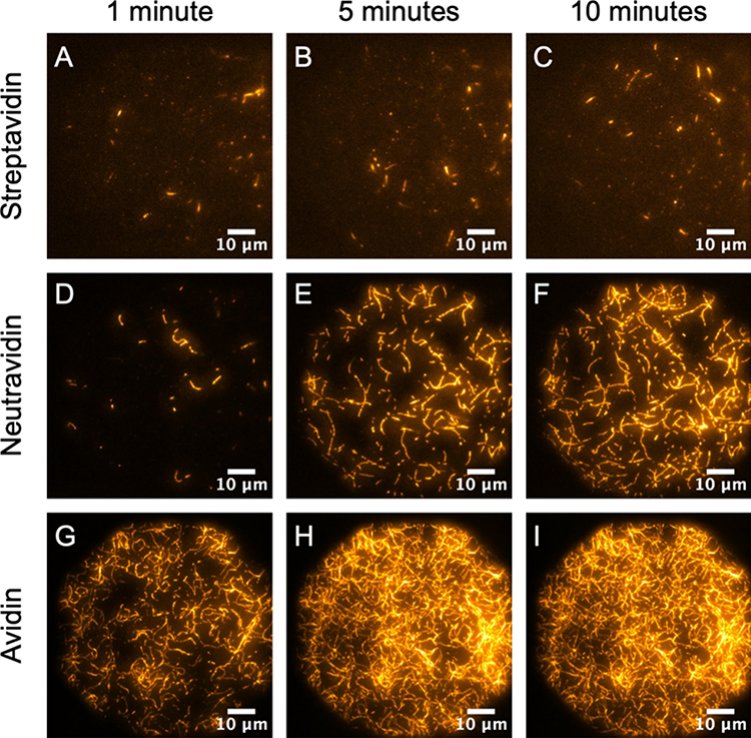
Time-dependent deposition of F-actin filaments in low salt (50 mM KCl) as a function of streptavidin, neutravidin, and avidin linkers. All images were collected with 0.1 mol % biotinylated SLBs on glass in the TIRF illumination mode. (A, D, G) 1 min; (B, E, H) 5 min; (C, F, I) 10 min.

**Figure 6:**
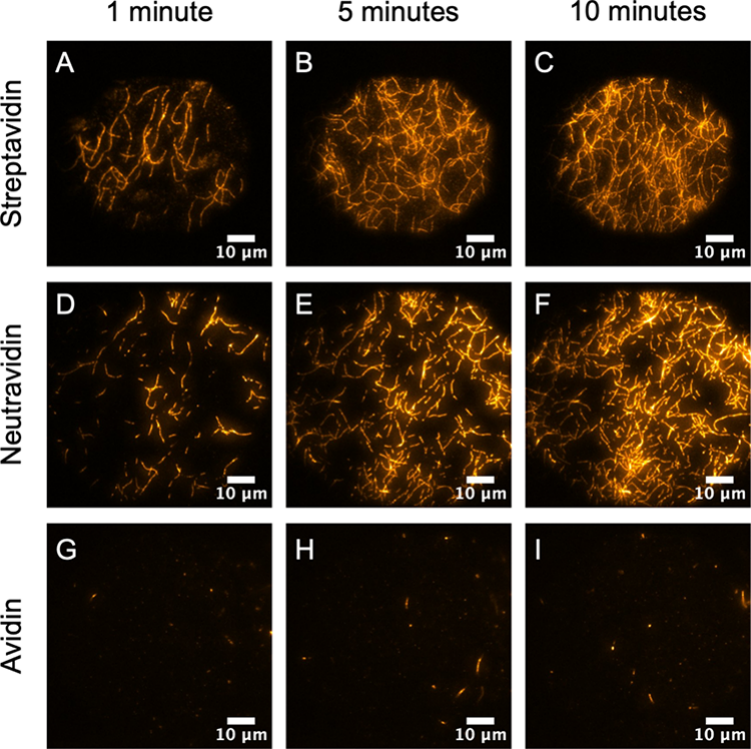
Time-dependent deposition of F-actin filaments in high salt (1 M KCl) as a function of streptavidin, neutravidin, and avidin linkers at 1 min (A, D, G), 5 min (B, E, H), and 10 min (C, F, I). All images were collected with 0.1 mol % biotinylated SLBs on glass in the TIRF illumination mode.

The dynamic process observed during individual filament adhesion suggests that the ionic strength should play an important role in the kinetics of layer deposition. Thus, we tested the three avidin-family linkers in the presence of both high and low salt concentrations (50 mM and 1 M KCl) and tracked the MAC layer formation over time. The results are shown in Figures 5 and 6.

At a low salt concentration (Figure 5), F-actin exhibits a distinct deposition rate for each of the three linkers. For streptavidin (Figure 5A–C), a very small number of short filament segments (<5 μm) adhere to the bilayer shortly after F-actin addition, but few new filaments adhere over time. The electrostatic repulsion between streptavidin and F-actin is generally large enough to prevent slow deposition and filament extension but not large enough to completely abolish filament association with the bilayer. Although neutravidin appears relatively unaffected by salt over long time scales (Figure 4), images collected over shorter time intervals uncover the impact of electrostatics (Figure 5D–F). At pH 7.5, neutravidin bears a slightly negative charge. Apparently, the small repulsive charge is just large enough to initially retard F-actin binding. However, it is not large enough to prohibit full filament extension and adherence. After a 10-min incubation period, maximum filament density is achieved. For avidin (Figure 5G–I), filaments adhere to the membrane quickly and form a packed layer that grows slightly more dense over time. Overall, it reached saturation rapidly. We attribute avidin’s accelerated deposition to the very strong electrostatic attraction between the linker and the F-actin that is not effectively screened in 50 mM KCl. Thus, the steric hindrance of the glycosylation sites is readily overcome.

At a high salt concentration, we observe a converse trend (Figure 6). Screening the repulsive interaction between the negatively charged streptavidin and F-actin permits the biotin tags to reach their streptavidin target quickly (Figure 6A–C). This leads to the appearance of extended and horizontally bonded strands shortly after F-actin addition, and the surface-bound density grows slowly over time. As would be predicted from electrostatics, the neutravidin deposition rate appears to be slightly enhanced (Figure 6D–F) compared to its low-salt counterpart. This is presumably due to a slight decrease in the innate repulsive interaction between neutravidin and F-actin. As with low-salt conditions, the existing repulsion is not large enough to prohibit full filament extension and adherence. Avidin, however, displays little binding over the entire time course (Figure 6G–I). We presume this arises from the concerted effects of reduced electrostatic attraction and the steric effect of glycosylation that hinders access of F-actin to the biotin-binding sites.

Because MACs and multi-MACs have potential utility in variable salt environments, the full range of electrostatic effects pertaining to layer formation needs consideration. To make informed decisions on the type of linker to be employed, awareness of both the achievable filament densities and the associated deposition time is important. As depicted in Figure 4, actin filaments have the potential to quickly saturate available linker sites when neutravidin or avidin is employed in the presence of a low salt concentration or when streptavidin or neutravidin is employed in the presence of a high salt concentration. However, the rate of deposition is notably different for each scenario; thus, the amount of time needed to achieve saturation varies. For streptavidin in the presence of high salt and neutravidin in both high and low salt, ∼10 min is required for filaments to saturate the linker sites. However, for avidin, in the presence of low salt, filaments saturate all available linker sites within minutes.

### Multi-MAC Formation

Multi-MAC creation follows an iterative (A-wash-B_*n*_-wash)_*N*_ algorithm. After priming the biotinylated lipid bilayer with a saturating concentration of linker (A), biotinylated filaments injected into the solution attach to the linker and gradually begin forming an interspersed and networked layer that leaves a significant fraction of the lipid bilayer exposed to the bathing solution. In an effort to precisely control and monitor filament deposition and layer formation processes, we inject filaments in a series of aliquots, or layers (*n*), at a concentration below saturation. After injecting multiple aliquots, enough filaments deposit to form a fully saturated tier (B_*n*_).

**Figure 7:**
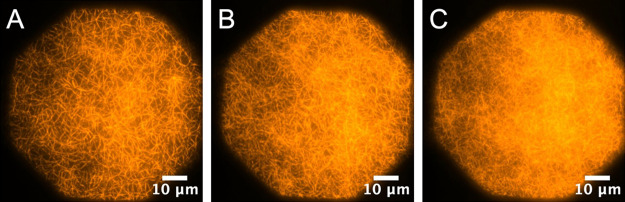
(A) Single-tiered MAC. (B) Double-tiered MAC. (C) Triple-tiered MAC.

Following saturation, a chamber rinse (5–10× chamber volumes) removes residual filaments, and a second aliquot of linker is added to coat the open biotin moieties on the exposed F-actin filaments. This primes the top surface of the deposited filaments for cross-linking with additional F-actin and initiates a new tier. After the residual linker is washed away, actin filaments are again added to the chamber in small aliquots, which results in additional deposition. The second layer reaches saturation once all accessible linker sites are occupied. Figure 7 shows sequential images in the multi-MAC construction process. As can be seen, as more tiers are added, the high filament density obscures the full identification of individual polymer strands.

Figure 7 shows the appearance of the resulting multi-MAC following full deposition (i.e., saturation) for a single tier (Figure 7A), an *N* = 2 multitiered MAC (Figure 7B), and an *N* = 3 multitiered MAC (Figure 7C) in the presence of a low-salt buffer (50 mM KCl) using avidin as the linker. The biotinylation density within the lipid bilayer is ∼0.1 mol %, the injected linker concentration is 0.2 μg/mL, and the mole fraction of biotinylated g-actin monomers incorporated into the filaments is 1:6 (17%). We employ this relatively high level of biotinylation in both the F-actin filaments and the lipid bilayer to create a large number of anchoring points. In turn, this creates a strong mechanical connection to the bilayer and between adjacent MAC tiers. Saturated area densities similar to that shown in Figure 7 also appear at lower percent biotinylation (e.g., 0.01 mol % in the lipid bilayer) and at lower concentrations of added F-actin.

For those combinations of salt concentration and linker type that facilitate filament deposition, we note that prior to saturation, a large majority of filaments adhere to the surface, and only a few remain in solution. That is, if the number of filaments injected is not sufficient to completely fill the surface binding sites, all free filaments will settle and bind to the lipid surface over time with a fully horizontal orientation. After repeated additions, F-actin shows diminished attraction for the linker-primed bilayer, or linker-primed MAC tier, and the rate of filament binding slows due to the occupation of available biotin binding sites. When saturation occurs, a majority of the filaments remain in solution above the surface. Filaments that do bind attach with only a portion of their contour length. In the TIRF mode, this gives the appearance of short filaments. The remaining unbound but tethered portions waver freely above the MAC (see the Supporting Information, Video S5).

**Figure 8:**
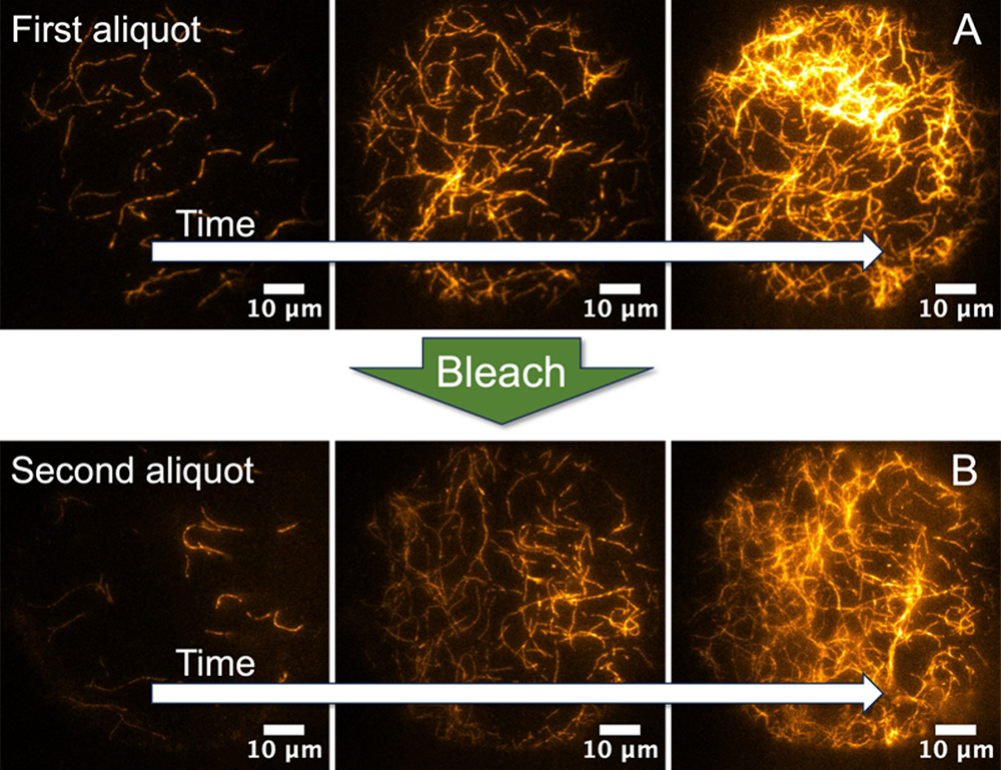
Bleach and measure protocol. After F-actin deposition saturates (A), the layer is photobleached before the addition of the next aliquot (B). This allows for large filament densities to be measured by the SOAX algorithm.

As actin deposition increases, the filament density grows too large for SOAX image processing to distinguish individual filaments between separate MAC tiers or even filaments within an individual sublayer. For this reason, we implement a bleach and measure protocol (Figure 8) to assist with the measurement of filament density as successive aliquots of actin are added (*n*) and new tiers (*N*) are cross-linked.

After a sublayer (*n*) fully deposits (Figure 8A), an image is obtained, and SOAX analysis is performed to determine the filament density. We then photobleach the initial layer of filaments until all filament fluorescence disappears. This creates a low fluorescent background and enables the subsequent layer of filaments (Figure 8B) to be visualized and processed by the SOAX algorithm without a contribution from the previous layer. This process repeats for each separate aliquot (*n*) of filaments added within each tier (*N*) of the multi-MAC. The same bleach and measure protocol applied between separate aliquots within an individual MAC layer is applied when adding a new tier of filaments (i.e., following linker priming).

**Figure 9:**
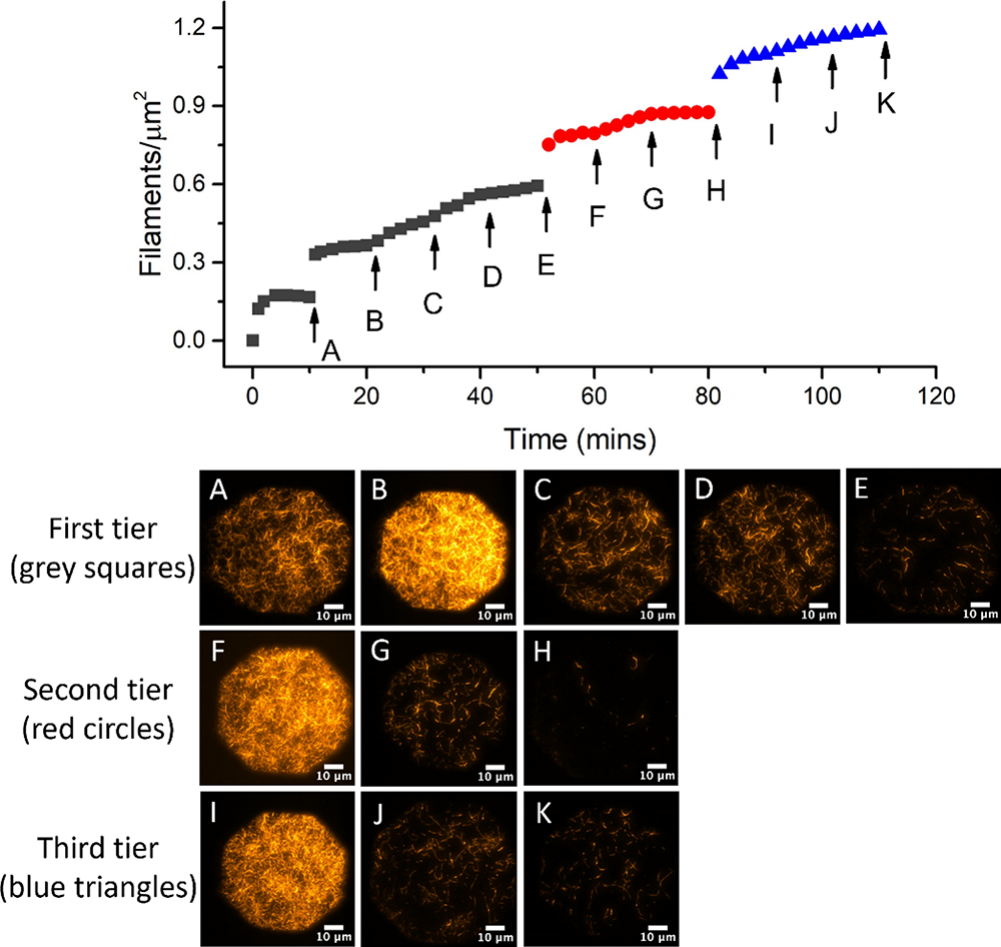
Filament density as a function of time and addition of actin filaments (arrows A–J) to a 0.1% biotinylated lipid bilayer using avidin under conditions of low ionic strength. First-tier filaments (gray squares) are directly attached to the bilayer. Red circles and blue triangles correspond to the second and third tiers, respectively. Images (A–K) correspond to arrows, where each image is obtained immediately prior to the bleaching and addition of new filaments (see text). E, H, and K correspond to single-, double-, and triple-MAC images shown in Figure 7A–C.

Using this bleaching procedure, the filament density of each layer can be determined individually. Thus, the total filament density of a multitiered MAC can be measured using SOAX. Results from a multilayered (*n*) and multitiered (*N*) MAC are shown in Figure 9. We added F-actin filaments in successive aliquots (*n*), as indicated by each discrete increment in filament density. Initiation of new cross-linked tiers (indicated by a change in color) typically causes a large density increase. The jump between A and B in Figure 9 arises from a disproportionally large filament deposition and not the initiation of an additional tier. New tiers (i.e., linker additions) are initiated prior to time points A, F, and I in Figure 9, with the most significant contribution to overall filament density occurring within the first tier. The filament density increase contributed by successively layered aliquots (n) generally decreases as available linker sites become filled. The fully saturated tiers labeled E–K correspond to the total filament densities displayed in Figure 7A–C.

We attempted to estimate the thickness of a saturated filament tier (AB_*n*_) by analyzing intensity changes upon scanning the TIRF incidence angle. However, the intensity changes resulting from a single actin tier (AB_*n*_) appear identical to the intensity changes arising from a fluorescently labeled lipid bilayer (i.e., ∼5 nm thick). This similarity indicates that a single tier of filaments occupies only a very thin region above the lipid bilayer surface with a thickness that remains well below the axial spatial resolution of our TIRF system (i.e., 250–500 nm).

Individual actin filaments and actin bundles have been measured by atomic force microscopy to be 8 and 16 nm in diameter, respectively.^49^ Biotin that is bound to streptavidin in opposing binding pockets can extend out to a distance of ∼2.5 nm.^60^ Thus, we estimate the first saturated F-actin tier to have a nominal height above the bilayer of 10.5–18.5 nm (assuming no bundling). Similarly, the second actin tier appears to be of a similar structure, which would increase the thickness of the combined tiers by another ∼18.5 nm. Thus, theoretically, two F-actin tiers form a porous network that occupies up to a 37-nm section above the lipid bilayer. Three F-actin tiers occupy less than 56 nm, etc. Indeed, even the theoretical distance estimated for the three tiers lies well below the measurable axial resolution of our system. Thus, we conclude that multi-MACs are very thin. This feature, coupled with the porosity of the structure, possesses many potential advantages for nanopore sensing and other applications where access to the underlying lipid bilayer is important.

### Impact of Foundational MAC on Diffusion

Many lipid bilayer applications, such as ion channel electrophysiology, nanopore sensing, or liposomal drug delivery, depend on the fluidity of the lipid. Translational diffusion is necessary for the aggregation and formation of ion channel proteins from lipid-bound monomers, transmembrane transport kinetics, and the lateral movement of species to any targeted molecule within the bilayer. However, the presence of an MAC, with its numerous bilayer attachment points and various electrostatic interactions, might alter this lipid dynamic. In an effort to characterize the impact of MACs, we performed FRAP on fluorescently labeled lipid bilayers coated with a single-tiered, nonfluorescent MAC (i.e., no rhodamine-labeled moieties).

**Figure 10:**
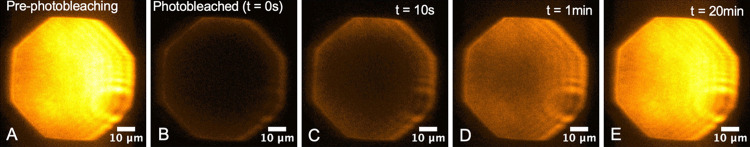
FRAP in a lipid bilayer with a tethered nonfluorescent single-tiered MAC. (A) Unbleached bilayer. (B) Bleached bilayer. (C–E) Fluorescence recovery from unbleached lipids initially located outside the illuminated area.

Figure 10 shows a sequence of FRAP images under approximate uniform illumination acquired in the TIRF mode. The recovery intensity is summed over a circular region with a diameter that approximates the octagonal diameter of the illuminated region (∼67 um). As can be seen, the original fluorescence intensity is recovered over the time scale of minutes.

Fitting the recovery curve to a two-component model provides diffusion constants, fractional values for each component, and a value for the immobile fraction (eq 1),^61^
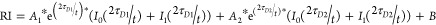
1where *A*_1_ and *A*_2_ are the fractional values of the two diffusing components, τ_*D*1_ and τ_*D*2_ are the characteristic diffusion times (τ_*D*_ = *w*^2^/4*D*), *w* is the radius of the illuminated area, *D* is the diffusion coefficient, *I*_0_ and *I*_1_ are zeroth and first-order modified Bessel functions of the first kind, and *B* is the immobile fraction. Fitting a two-component diffusion model to the integrated fluorescence recovery data results in a good fit.

**Figure 11:**
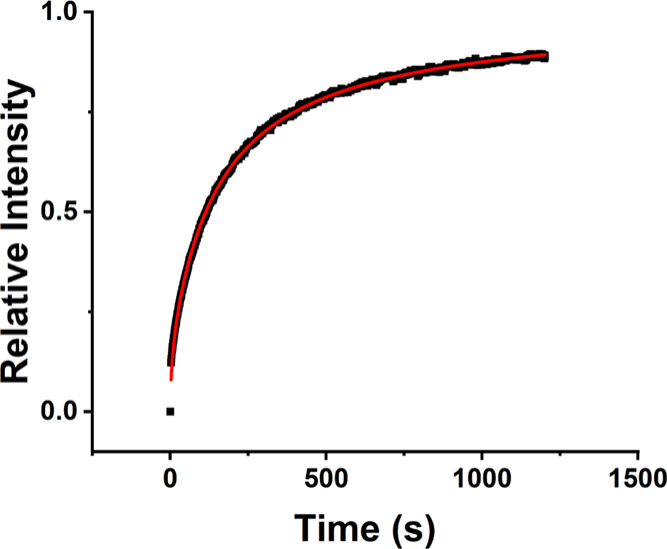
Normalized recovery curve from the spatially integrated intensity fit with eq 1.

Figure 11 shows an example of the recovery curve from the integrated image intensity data (black) with the fit overlaid (red). For all surfaces tested, only one diffusive component is prevalent (>90%), and the immobile fraction (*B*) is small (<1%). Thus, we summarize trends arising from the majority component only. For a lipid membrane without biotinylated lipids, a linker, or a MAC, the analysis produces a diffusion constant of *D* = 2.5 μm^2^/s. This serves as a reference for comparison to the MAC-coated bilayers.

**Figure 12:**
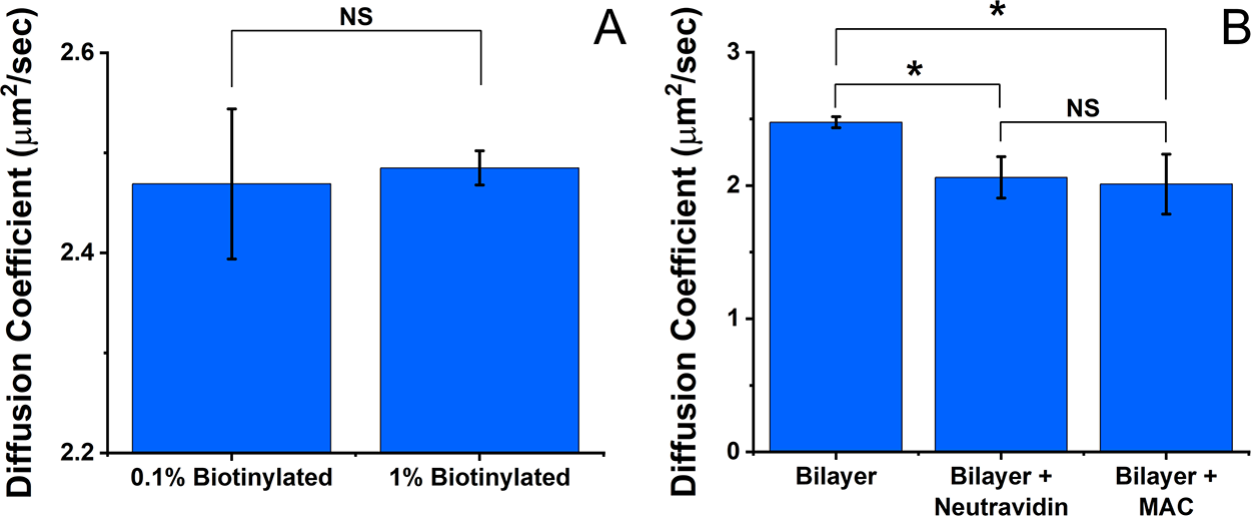
(A) Lipid bilayers containing 1 and 0.1% biotinylated lipids possess similar diffusion coefficients. (B) The average diffusion coefficient for the bilayer is 2.5 μm^2^/s, and the foundational linker alters diffusive motion of the lipid the most. Error bars: SEM of 3–5 measurements. Two-sided student *t* test: *P* < 0.08, NS *P* > 0.08.

Altering the concentration of biotinylated lipids in the bilayer has the potential to influence the rigidity and porosity of the linked F-actin layers. In addition, at high concentrations, the number of anchor points within the membrane might also impede the lateral diffusion of bilayer-incorporated molecules, which is particularly important for nanopore biosensors. However, our observations indicate that biotin anchor concentrations (up to 1 mol %) impact the rate of lipid diffusion very little (Figure 12A). Bilayers with 1 or 0.1 mol % biotin are statistically indistinguishable. However, a 1% biotinylated bilayer with neutravidin linker lowers the observed diffusion constant slightly (Figure 12B). Addition of a single-tier MAC does not retard diffusion further (Figure 12B). Apparently, the most significant influence on lipid diffusion arises from the linker, not the F-actin filaments. Overall, however, the lipids remain in a highly fluid state. Furthermore, both the relative fraction of the two diffusion components, as well as the immobile fraction, change very little after the construction of the single-tiered MAC. We presume that the presence of additional F-actin tiers has a negligible impact on lipid diffusion.

### Multi-MAC Resistance to Shear Stress

Numerous bilayer applications require tolerating shear stress. Liquid moving around drug-loaded liposomes or nanoparticles can impact stability and delivery efficacy when placed in the bloodstream. In addition, biological nanopore sensors can require implementation in a fluid flow cell. Thus, bilayers need to withstand exposure to a solution exchange. Multi-MAC coverings provide a potential remedy for situations where shear stress can disrupt the integrity of the bilayer.

**Figure 13:**
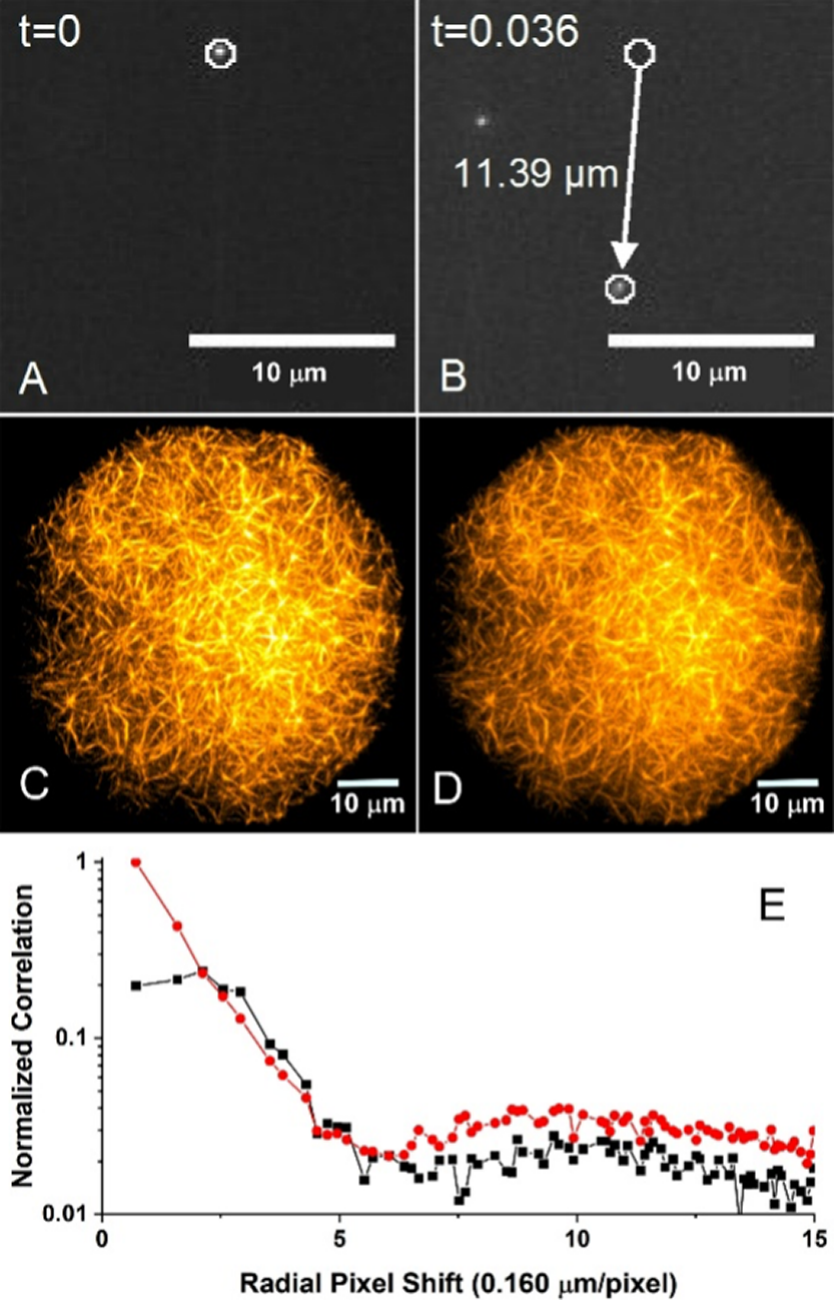
Multi-MAC durability under fluid flow. (A, B) Laminar flow velocity at the interface measured by linear displacement of fluorescent beads (∼315 μm/s). Multi-MAC filaments before (C) and after (D) exposure to a Laminar flow. (E) Image correlation analysis. Autocorrelation of C (red) and cross-correlation of C and D images (black) reveal an inconsequential shift of the filaments after a 5-min exposure to fast fluid flow.

We tested the durability of multi-MAC structures by varying the flow velocity of the bathing solution in a laminar flow cell while probing for changes in the multi-MAC structure. Figure 13A,B shows two TIRF-illuminated frames from a fast time-lapse video. The change in position of injected 40 nm fluorescent beads between frames allows the Laminar flow velocity of the solution in contact with the multi-MAC interface to be estimated. Our apparatus generates flow velocities from 50 to 325 μm/s. However, even the highest flow velocity did little to disrupt the interwoven structure, or filament positions of the multi-MAC. Figure 13C,D shows the filamentous structure before and after exposure to a fluid flow of ∼315 μm/s. Image correlation analysis (Figure 13E) provides a means of assessing more subtle conglomerate displacement of filaments, which elude direct visual inspection. In comparison to the benchmark autocorrelation of Figure 13C that characterizes complete immobility (red), the cross-correlated images of Figure 13C,D reveals a slight shift in the overall structure of the multi-MAC. However, the net displacement is miniscule. The peak of the cross-correlation curve indicates average filament displacement on the order of the diffraction-limited resolution of the camera (∼2.5 pixels, ∼400 nm). This apparent structural tolerance to shear stress suggests that multi-MACs are suitable for applications involving fast and repetitive rinse cycles common to various sensing applications.

## Conclusions

This work describes the design and construction of a multitiered lipid bilayer support structure that is composed of stabilized actin filaments which are cross-linked with each other via avidin family proteins and bonded to lipid headgroups of the underlying bilayer. We refer to the novel structure as a multi-MAC. The multitiered nature of multi-MACs has the potential to enhance the mechanical and electrical properties of various lipid bilayer structures in unique ways. Applications include bonded or tethered SLBs, unsupported lipid bilayers, liposomes, and vesicles of various curvature and size.

To address design and construction issues, we explore the impact of electrostatics on the deposition rate, filament density, and cross-linked structure. These features are observed directly on model planar bilayers using TIRF and widefield fluorescence microscopy. The varied behavior of linkers and F-actin can be explained theoretically by the isoelectric points and the molecular structure of each component. We also detail the structure and deposition kinetics of F-actin layers as a function of the solution ionic strength. In an effort to understand and visualize the assembly process, we purposefully employ a filament-by-filament layering technique, whereby F-actin is deposited in an incremental fashion. However, this stepwise approach is not fundamentally necessary for successful multi-MAC construction. Individual tiers of F-actin can be deposited more quickly by further optimizing solution conditions and adding F-actin filaments in larger quantities.

We also demonstrate that the foundational tier of a multi-MAC that is chemically linked to lipid headgroups only slightly alters lipid diffusion properties. This is significant for applications that need to maintain the lipid bilayer fluidity (e.g., membrane protein aggregation and nanopore sensor insertion).

Lastly, we explore the ability of the multi-MAC support structure to withstand shear stress. This is particularly relevant for nanopore sensing and liposome cargo delivery applications. Our flow cell results suggest that a multi-MAC structure is highly robust and able to withstand high-solution velocities across the surface.

Although the impetus for developing multi-MAC structures arises from the desire to improve nanopore sensing applications, the general methodology described herein can be applied to a large number of bilayer systems where extra support and mechanical resistance to stress is needed.

## Supporting Information Available

Additional experimental details (PDF)
Dynamics of F-actin binding to lipid bilayer surface (MPG)
Visualizing anchored and vertically oriented filaments (MPG)
Electrostatic repulsion of full-length filaments (MPG)
Salt concentration controls filament deposition (MPG)
MAC construction and F-actin saturation (MPG)

## Supplementary Material

mt3c01267_si_001.pdf

mt3c01267_si_002.mpg

mt3c01267_si_003.mpg

mt3c01267_si_004.mpg

mt3c01267_si_005.mpg

mt3c01267_si_006.mpg
