# Supplementary material for: Design and Construction of a Multi-Tiered Minimal Actin Cortex for Structural Support in Lipid Bilayer Applications
Source: ACS Appl Bio Mater. 2024 Mar 1;7(3):1936–46. doi: 10.1021/acsabm.3c01267 (PMC10951949; doi:10.1021/acsabm.3c01267)
Supplement: Supplementary file 1 — mt3c01267_si_001.pdf [file mt3c01267_si_001.pdf]

Supporting Information

Design and Construction of a Multi-Tiered Minimal Actin Cortex (Multi-MAC) for Structural Support in Lipid Bilayer Applications

Amanda J. Smith, Theodore R. B. Larsen, Harmony K. Zimmerman, Samuel J. Virolainen, Joshua J. Meyer, Lisa M. Keranen-Burden, Daniel L. Burden*

Wheaton College Chemistry Department
501 College Ave, Wheaton, IL 60187
* Corresponding author: daniel.burden@wheaton.edu

Table of Contents

**Video S1**	**S1**
**Video S2**	**S1**
**Video S3**	**S1**
**Video S4**	**S1**
**Video S5**	**S1**

Video S1: Dynamics of F-actin binding to lipid bilayer surface. Filaments adhere in a gradual end-to-end sequence over time. The timescale of the adhesion process varies depending on linker type and ionic strength and can range from a few seconds to multiple minutes.

Video S2: Visualizing anchored and vertically oriented filaments. Orthogonally oriented F-actin filaments are partially bound to a lipid bilayer with a streptavidin linker and 50 mM salt concentration. The mode of imaging is slowly changed from TIRF to widefield by lowering the coated coverslip and simultaneously changing the angle of the incoming laser. The image plane is fixed. This technique permits the portion of filaments extending above the surface to be visualized. Only the ends of filaments bound to the surface can be seen in the TIRF imaging mode. However, the corresponding unbound portions of filaments can be seen extended in orthogonal direction away from the surface during the transition to the widefield mode.

Video S3: Electrostatic repulsion of full-length filaments. With no added salt, F-actin binding to a streptavidin-coated bilayer surface is sparce due to a large electrostatic repulsion. Here, the mode of imaging is changed from TIRF to widefield by lowering the coated coverslip and raising it up again, while simultaneously changing the angle of the incoming laser beam. The image plane remains fixed. This technique permits visualization of unbound filaments that are highly repelled from the surface (without any anchor points). Entire unbound filaments can be seen a large distance away from the surface.

Video S4: Salt concentration controls filament deposition. Time-lapse TIRF video of avidin-coated surface involving continuous fluid exchange that changes the salt concentration over time. Low salt concentrations enable F-acting binding. High salt concentrations prohibit binding due to the steric interference from avidin glycosylation.

Video S5: MAC construction and F-actin saturation. In the TIRF mode, surface saturation gives the appearance of short filaments binding to the surface because filaments bind with only a portion of the available contour length.
